# Concept analysis: Community-based postnatal care

**DOI:** 10.4102/curationis.v46i1.2423

**Published:** 2023-12-04

**Authors:** Katekani J. Shirindza, Thivhulawi Malwela, Maria S. Maputle

**Affiliations:** 1 Department of Advanced Nursing Sciences, Faculty of Health Sciences, University of Venda, Polokwane, South Africa

**Keywords:** concept analysis, community-based, postnatal care, early discharge, primary caregivers, postnatal woman, newborn

## Abstract

**Background:**

Community-based postnatal care is a valuable resource in the provision of maternal and neonatal care, specifically outside the hospital environment. However, its application in maternal and neonatal care is not clearly documented in relation to the rendering of services by primary caregivers.

**Objectives:**

This study clarifies the concept of ‘community-based postnatal care’ by using the concept analysis method.

**Method:**

To analyse the concept, relevant literatures were reviewed and analysed using the Walker and Avant method, namely, selecting a concept, determining the purpose of analysis, identifying all uses of the concept, defining attributes, identifying a model case, identifying borderline, related and contrary cases, identifying antecedents and consequences and identifying the empirical referents. Characteristics that repeatedly appeared throughout the literature were noted and categorised.

**Results:**

It was established from the concept analysis that ‘community-based postnatal care’ was complex and experienced ethnically. The analysis included that primary caregiver participation was based on home-levelled-skilled care, community participation and mobilisation, linkages of health services and community stakeholders. The attributes were influenced by antecedents and consequences.

**Conclusion:**

The empirical referents of community based can be integrated within the midwifery guidelines to measure the concept. When concepts are understood, self-care on early detection, early management and referral during early postnatal care will be enhanced.

**Contribution:**

The results of this study will foster independence, confidence and a respectful relationship between primary caregivers and the health care facility staff. The results are expected to guide future research and enhance community-based postnatal care in midwifery practice.

## Introduction

A concept can express the same idea but utilising different sets of words (Tofthagen & Fagerstrom 2010). The aim of concept analysis is to specifically give clarity to the meaning of concepts, similarities and identify various considerations for measuring the concept (Falan 2010) as cited in Maputle and Hiss (2013). Concept clarification is an important step in developing the knowledge that can be useful in with continuing provision postnatal care; hence in this article, concept analysis was conducted after the research that was conducted in the three selected districts of Limpopo province, South Africa. The study explored the experiences of primary caregivers on early postnatal care at home. Primary caregivers are the people who took care of a newborn and a mother post-discharge or someone who is sick (Richards & Schmidt 2011) as cited in Shirindza, Malwela and Maputle (2021). In this study, a primary caregiver is any family member who takes the primary responsibilities to postnatal woman and her baby immediately after discharge from the delivery facility and continue with the routine postnatal care at home (Shirindza et al. 2021). The primary caregiver can be a guardian or a close relative who provides postnatal care activities from delivery until 6 weeks period.

The research was conducted in four phases: phase 1 – empirical, phase 2 – concept analysis, phase 3 – model development and phase 4 – model validation. For empirical phase, a qualitative, explorative, descriptive, contextual and inductive research approach was followed. The sample consisted of 20 primary caregivers from three different ethnic groups of the Limpopo province. Data collection was obtained from unstructured interviews and analysed through Tesch’s open coding method (Creswell & Cresswell 2018). The findings of the main study were the knowledge and skills of providing postnatal care, facilitators regarding the continuity of postnatal care and the barriers related to the continuity of postnatal care. After a description of the experiences of primary caregivers and synthesis of the results from the empirical data, the concept ‘community-based postnatal care’ emerged as a central concept. This paper focused on phase 2-concept analysis.

## Background

The situation in hospitals and clinics is such that protocols are adhered to as laid down by the World Health Organization (WHO) (2015) guidelines and procedures. Postnatal women and newborns were generally discharged from the health facilities within 6 h in the absence of complications. However, the pair would be at risks of developing complications within 24 h, 48 h and 72 h, while at times this might extend beyond the duration mentioned. In many resource-constrained settings, there are trained community health workers (CHWs) who are often the first point of contact between communities and the health care system (Wouters et al. 2012). They play a vital role in conveying essential health information in a culturally sensitive manner, empowering community members to make informed decisions and improving community-level access to essential health services (Lewin et al. 2010).

Primary caregivers continued with postnatal care at home, though they were not trained, involved and were not participating when the pregnant woman attended antenatal care. A study by Orjingene and Morgan (2020) pointed, there was a high number of maternal deaths following delivery. In 2008, 342 900 women died globally and six nations such as Nigeria, Ethiopia, Tanzania, Kenya, Zimbabwe and Uganda contributed to more than 50% of these deaths. The first week of life particularly vulnerable period, with 75% of neonatal deaths occurring during this time (Bee, Shiroor & Hill 2018). In South Africa, the maternal, neonatal and child deaths are unacceptably high, ranging from 310 deaths per 100 000 live births, neonatal mortality rate of 14 deaths per 1000 live births and child under-five years mortality rate of 56 deaths per 100 000 live births (Mabaso, Ndaba & Mkhize-Kwitshana 2014). Most of the deaths were high in the Free State, Limpopo, KwaZulu-Natal, Eastern Cape and lower in Mpumalanga, North-West, Northern Cape, Gauteng and Western Cape provinces (Mabaso et al. 2014). The World Health Organization reports that 830 women across the world die each day from pregnancy-related complications and during delivery (Mojekwu & Ibekwe 2012). Most of the maternal and neonatal deaths are a result of following the pre-discharging period. The end results of early discharge of both the women and newborns postnatally resulted to a burden to primary caregivers of which some were not trained on health issues following care after delivery.

The study was conducted among 20 primary caregivers from three different ethnic groups in Limpopo province: namely Xitsonga, Vhavenda and Northern Sotho. Primary caregivers understood cultural norms and the societal emotional support to mothers (Guta et al. 2018). They were using indigenous skills, practices and knowledge when caring for postnatal women and babies. This care was complex with potential risks to the woman and the neonate.

Community health workers are available at primary health care facilities; however, it was not clear whether they work collaboratively with primary caregivers to provide continuity postnatal care. This strategy had been implemented across multiple service delivery platforms, and good progress has been made in reducing maternal and newborn deaths (Gogia & Sachdev 2016; Hodgins, McPherson & Kerber 2017). Provision of community-based postnatal care following childbirth has been found to be one of the key recommendations of the Ending Preventable Maternal Mortality initiative and the Sustainable Development Goals (SDGs) to reach the global goal of reducing maternal mortality (70 maternal deaths per 100 000 live births) (Jolivet et al. 2018; UN-DESA 2017).

There has been a lack of agreement on the definition regarding community-based postnatal care so far, even though several studies have examined community-based postnatal care experiences. It is important to clarify community-based postnatal care as a concept so that its theoretical and practical aspects can be better studied. This in turn could lead to the development of research tools for measuring it. For this reason, the researchers analysed the aspects of community-based postnatal care by using the concept analysis method found in Walker and Avant (2013). Concepts analysis is one of its own methods used to derive precise theoretical and operational definitions of certain words, terms or symbols by clarifying their constituent properties (Park & Park 2014). A literature review was used to define the concept ‘community-based postnatal care’. In addition, the antecedents, attributes, consequences and empirical referents emerged during the analysis of the concept. Finally, model, borderline and contrary cases are provided to better understand the concept of community-based care.

## Problem-statement

Mother and newborn were discharged within 6 h post-delivery, and CHWs were available at the primary health care facilities; however, their role in empowering primary caregivers on basic postnatal care to continue with care was not known. Primary caregivers would be using their indigenous knowledge, skills and practices to continue with the provision of postnatal care, some of which could be harmful. A synthesis of results from the empirical data on the experiences of primary caregivers revealed the core concept as the need for ‘community-based postnatal care’. The concept ‘community-based care’ has frequently been used in literature; however, it was found that no consensus has been reached about its meaning and its use in postnatal care. This article used Walker and Avant’s (2013) method of concept analysis as a framework to analyse community-based postnatal care.

## Aim of the study

The aim of the study was to conduct concept analysis of community-based postnatal care, to clarify meanings of similarity and identify multiple considerations for measuring the concept. Hence the research question addressed in this article was: ‘What is the meaning of community-based care in the context of postnatal period’?

## Research design and method

### Research design

Concept analysis was conducted to obtain the operational definition and to define attributes, antecedents and consequences of the concept ‘community-based care’. In addition, a model case, a borderline case and a contrary case were constructed to achieve conceptual clarity (Malusky 2005; Sun & Knobf 2008) as cited in Maputle and Hiss (2013). Concept analysis is used to examine and describe a concept and its application. The objective is to understand clearly what all about the concept is, to such an extent that the concept should be clear and distinct, defined and well differentiated from other concepts and should be applicable to the world and be appropriate to the specific context (Chabeli & Muller 2004).

The following are the specific steps to follow when conducting concept analysis as derived from Walker and Avant (2013):

- Select the concept to be analysed
- Determine the aim and purpose of the study
- Identify the uses of the concept
- Determine the defining attributes of the concept
- Construct model case, borderline case and contrary case illustrating the concept
- Identify the antecedents and consequences of the concept
- Identify empirical referents.

### Steps used in concept analysis

- Selecting a concept of interest, relevance, importance and usefulness
  The findings of the study ‘Experiences of early postnatal care by primary caregivers at the three selected districts of Limpopo Province was conducted’ (Shirindza et al. 2021). The experiences of primary caregivers during the postnatal care period revealed the major concept as ‘community-based postnatal care’. Community-based care was found to be the central idea or event and all the other categories and attributes were contingent upon it.
  Community-based postnatal care was selected as a core concept in this study because it was found to be the central needed intervention, and all the other ideas are related to it. Walker and Avant (2013) recommended using dictionaries, thesauruses and any possible literature to identify the use of the concept. The word ‘community-based postnatal care’ could not be found in dictionary. Thus, researchers searched for ‘community-based’ in general. According to Cambridge English Dictionary (Hoadm 1993), community based was used to describe an activity that is organised and takes place locally. Community is the people living in one area who are considered as a unit because of their common interests, social group or nationality (Cambridge English Dictionary 2022). A community is sometimes used to describe a society in general. It is sometimes referred to ‘a group of people who have the same interest or religion’. The suffix ‘based’ is used to form adjectives showing the ‘main place or area in which something or someone works, lives or does businesses’. It means ‘working or doing business in a particular place, or in a particular way’.
  In this context, early postnatal care refers that primary caregivers can provide postnatal care post-discharge and at home, to detect and prevent the development of complications from the mother and the neonate. The study conducted by Mnisi, Peu and Meyer (2012) as cited in Guta et al. (2018) revealed the importance of training community members to prevent health challenges in the community, which is in line with the developed model (phase 3, not part of this manuscript).
- Aims of concept analysis
  The aim of concept analysis was to distinguish between ordinary and the scientific utilisation of the concept ‘community-based postnatal care’, which was necessary for concept clarification to add to the existing theory and to develop an operational definition (Walker & Avant 2013). The aim of concept analysis was to generate new ideas in the provision of early postnatal care at home and community context (outside of the health facility environment). Literature review was conducted to achieve the aims of analysis to identify the shared meaning of imaginative concepts. It further explored the use of the concept ‘community-based postnatal care’ within the context of community midwifery practice in the Limpopo province. The critical attributes of ‘community-based postnatal care’ as both the process and product had to be identified and the differences between the ‘connotation and reference’ distinguished (Smeltzer et al. 2008). The analysis determined the meaning of the concept ‘community-based postnatal care’ and its usefulness, applicability and effectiveness as a key concept to community-based postnatal care.
  The recommendations by Wilson (1963) as cited by Guta et al. (2018) reflected that an analyst determines who might use the concept and when, how and why it may be used as a way of determining the contexts within which to use the concept itself. Community-based postnatal care has different meanings and interpretations in various contexts both globally and nationally. In this study, the concept ‘community-based postnatal care’ was used to clarify and describe the meaning of community-based postnatal care within the context of early detection and preventing maternal and neonatal complications by primary caregivers at home. This context helped the development of a theoretical definition of the concept by clarifying the basic elements, structure and functions of the concept (Chinn & Kramer 2008; Walker & Avant 2013).
- Uses, characteristics and connotations of the concept.
  Community-based care is defined as a philosophical approach in which communities have an active role to participate in highlighting and addressing the issues that matter to them (Hoadm 1993). According to Sellers (1993), postnatal care is the care that is given to the mother and the baby immediately within 6 weeks after delivery. The South African Nursing Council (SANC 2014) defines postnatal care as the period that lasts 6–8 weeks, beginning right after the birth of the baby. According to WHO guidelines (2014), postnatal care is provided to both the mother and baby immediately after the birth of the baby for at least 24 h. World Health Organization (2014) defined postnatal care services as a constellation of preventive care, practices and assessments designed to identify and manage maternal and newborn complications during the first 6 weeks after birth. WHO (2014) recommended four postnatal visits to mothers and newborns to improve their survival. The recommended postnatal visits should be in the first 24 h, on the third day, between days 7 and 14 and 6 weeks of childbirth (Mon, Thinkhamrop & Bandit 2018). Fraser and Cooper (2010) define postnatal care as the care that is provided to the mother and the baby immediately after the expulsion of the placenta and membranes and continues until 6 weeks after the delivery of the baby.
  Therefore, in this analysis, ‘community-based postnatal care’ refers to the philosophical approach in which primary caregivers possess the knowledge and skills to provide early basic postnatal care at home. They have an active role and addressing the health issues of both the mother and her baby post discharge until 6 weeks period. Consequently, the primary caregivers should take full responsibilities to the lives of both the mother and the baby as stipulated by the SANC regulation (R2488), making the most difference to the health and life changes of mothers and newborns in the early stages of postnatal period. According to the Scope of Practice of a registered midwife (R.2488, as amended), the registered midwife is expected to attend to both the mother and baby at delivery and then hand over to the CHWs, then the primary caregiver to take over the active role of postnatal practices at the household level.
  The concept of ‘community-based care’ is frequently not well defined in dictionaries. As a provider of community-based postnatal care, primary caregivers are part of the community. Community-based care in the context of preventing maternal and newborn complications; therefore, it can be defined as home or community-level skilled care provided by the community, for the community with linkages to health facilities to avoid all forms of delay (Guta et al. 2018). According to the study conducted by Guta et al. (2018), the community is defined as the provision of skilled therapy services within a client’s own home or community with respect to the lifestyle of the client and the cultural and social characteristics of the client’s community. There are four models that relate to the current discussion, namely community as a setting, community as a target, community as resource and lastly community as agent. Community as a setting refers to the setting for interventions (Merzel & D’Affitti 2003). The setting is identified through the assessment, planning and management of community-based care activities for proper interventions. Therefore, the setting helps primary caregivers to detect complications that might arise during the provision community-based care services postdischarge. The second mode, which is the community as a target, relates to primary caregivers rendering postnatal care activities to woman and neonate at the home environment to prevent complications. A third model of community-based care is community as a resource, which is mostly used in health promotion services to enhance community ownership and participation for sustained health outcomes. This model can be applied to primary caregivers with information regarding community-based postnatal care in the communities. The primary caregivers within the community are the perfect fit to be trained on basic postnatal care activities to detect and prevent maternal and neonatal complications. The fourth model of community-based care is community as an agent. This affirms that primary caregivers need respect and recognition to have an adaptive, supportive and developmental capacities of communities. According to Steckler et al. (1993), communities provide resources for meeting the needs of community members on daily basis.
- Determining the defining attributes of community-based postnatal care
  According to Walker and Avant (2013) and Chinn and Kramer (2008), determining the defining attributes has been the ‘heart’ of concept analyses. The components that constitute the concept should be applicable to any situation in which the concept is located. Defining attributes is a process of clustering characteristics most frequently associated with the concept, which functions much like criteria for making differential diagnosis (Walker & Avant 2013). The defining attributes help in differentiating concepts from others similar or related to it. The defining attributes are the characteristics one sees repeatedly in the literature. In this study, the researcher reviewed the relevant literature and then noted and summarised the characteristics that appeared repeatedly. The characteristics were revised and modified based on the decisions by the authors. In this study, the defining attributes are the aggregates associated with the word ‘community-based postnatal care’ and ranked in order of importance (Walker & Avant 2013). From the uses, characteristics and connotations of community-based care, the following attributes were deduced.

#### Provision of home and community-level skilled care

Primary caregivers are not just family members but also the best in helping postnatal women and babies as they know and understand the local traditional customs within the community (Guta et al. 2018). The following are the activities that must be used by primary caregivers at the community level to both the mother and the newborn baby:

- Detection of danger signs in a newborn such as stopped feeding well, convulsions, fast breathing, severe chest indrawing, fever and yellow palms and soles.
- Health promotion interventions such as exclusive breastfeeding, avoidance of bathing until 24 h after birth, appropriate clothing to the baby and no separation to both mother and baby for bonding
- Health prevention interventions umbilical cord care, avoidance of harmful traditional substances such as cow dung to the cord stump
- Early referrals to the health care facilities in case of complications (WHO 2014).

The following are the activities that should be considered by primary caregivers to the mother for community-level care such as assessment of vaginal bleeding, uterine contraction, bowel functioning, any perineal wound, headache, fatigue, backache, perineal pain, breast pain, uterine tenderness, micturition and urinary incontinence as well as offensive vaginal discharges.

In this case, interventions are made available by primary caregivers where they are mostly needed to save the lives of the woman and baby, and these services are rendered by primary caregivers at the community level. The primary caregivers should be able to give regular report and feedback regarding the outcomes of the community-based postnatal care skills.

#### Community participation and mobilization

According to Guta et al. (2018), community (primary caregivers) assesses their own health needs and develops their own means of meeting the needs of postnatal women and babies after delivery. The community-based postnatal care coincides with a collaborative process between health care facilities and community stakeholders. The stakeholders engage with the health care facilities to address issues that might contribute to the enhancement of community-based postnatal care at the community level to sustain the health of both the women and babies after delivery. The process can be enhanced through the identification of primary caregivers within the community and involve them in taking care of both the women and babies after delivery at home. The primary caregivers should also be accessible to their clients to avoid problems of seeking emergency transport when faced with complications.

#### Linkages between health services and community health workers

The study conducted by Guta et al. (2018), on linkages between the health care facilities and the CHWs, brings together their thoughts and combines them to assist with the effective care of the women and babies after delivery. Postnatal women and their babies cannot survive alone at home when faced with postnatal complications but need immediate care from primary caregivers to prevent complications. The linkages can be accessed by deploying community health care workers to be the intermediate between health care facilities and primary caregivers.

### Case description and analysis of community-based postnatal care

Walker and Avant (2013) pointed that concept analysis to develop one or more model cases that represent a real-life example of the use of the concept that includes all the critical attributes of the concept. Walker and Avant indicated in the model an examination of additional cases that originated from Wilson’s model (1963). The cases are divided into borderline, related and contrary cases. The concept analysis method described by Walker and Avant (2013) uses case studies to clarify the concept and its attributes. A model case is an example case that depicts the concept with all of the defining attributes. A borderline case is a case that contains most but not all the defining attributes. Finally, an example of a contrary case is what is ‘not a concept’. According to Walker and Avant (2013), a model case is a real-life example of the use of the concept, which includes all the defining attributes of the concept. All the critical attributes of the concept ‘community-based postnatal care’ include the early detection of danger signs, health promotion to mother and baby and health counselling to the mother.

#### Model case and analysis

The following is a model case:

*Mrs Rahel is a 65-year-old woman who stayed with her daughter in law who gave birth to the new-born baby at the nearest health care facility. She was discharged after 6 h postnatally, Mrs Rahel needed to continue with postnatal care provision at home. Mrs Rahel once worked as a home-based carers at a district hospital. She started provided home-based postnatal care to her daughter in law. She assessed her to exclude postnatal potential risks from the day of discharge until the 6 weeks period. She worked with the mother and baby, by assessing to detect the danger signs in the baby like when the newborn was not feeding well, she advised on correct feeding practices. When the baby was too hot (risk of imminent convulsions, fast breathing, severe chest-in drawing, fever, had yellow palms and soles of feet. She would detect this early and refer to the relevant health facility.*

*She assisted the daughter in law in the promotion of health to the baby by encouraging exclusive breastfeeding, umbilical cord care, avoidance of harmful traditional practices, appropriate clothing to the baby and avoidance of bathing until 24 h. To the mother, she gave health education to the postnatal woman regarding the physiological process of recovery after birth and advice to report any health concern such as profuse blood loss, dizziness, faintness, palpitations and signs of vomiting and offensive vaginal loss. she assisted the postnatal woman with assessment of vaginal bleeding, uterine contraction, bowel functioning, bladder functioning, observation of healing of perineal wounds, fatigue, backpain, breast pain, uterine tenderness and the observations of lochia.*

*As she was working with the mother, she was transferring basic postnatal skills for her women and baby. Community-based postnatal care facilitates skill transfer through training, health education, counselling and referral where necessary. After referral to the health care facilities, the feedback was made a priority to herself and the primary caregivers within the community. She also knew that the community supported primary caregivers on community-based care based on their cultural norms.*

This model case demonstrates fully the attributes of community-based postnatal care, namely the provision of community-level skilled care, skills transfer and health education, counselling and referral to relevant health facilities.

#### Borderline cases and analysis

A borderline case contains some of the characteristics but not all the defining attributes (Walker & Avant 2013).

*Mrs Rahel once worked as a home-based career at a district hospital. She assisted the daughter in law in the promotion of health to the baby by encouraging exclusive breastfeeding, umbilical cord care, avoidance of harmful traditional practices, appropriate clothing to the baby and avoidance of bathing until 24 h. To the mother, she gave health education to the postnatal woman regarding the physiological process of recovery after birth and advice to report any health concern such as profuse blood loss, dizziness, faintness, palpitations and signs of vomiting and offensive vaginal loss. she assisted the postnatal woman with assessment of vaginal bleeding, uterine contraction, bowel functioning, bladder functioning, observation of healing of perineal wounds, fatigue, backpain, breast pain, uterine tenderness and the observations of lochia. As she was working with the mother, she was transferring basic postnatal skills for her women and baby.*

This model case demonstrates the linkages of communities and health facilities and community mobilisation but no provision of community-level skilled care. This is a borderline case as it did not comprise all of the critical attributes on the enhancement of community-based postnatal care.

#### Contrary case and analysis

A contrary case exhibits none of the defining attributes of community-based postnatal care (Walker & Avant 2013). The contrary case exemplifies the opposite of enhancement of community-based postnatal care, which leads to non-accomplishment of objectives. The following is an example of a case:

*Mrs Rahel is a 65-year-old woman who stayed with her daughter in law who gave birth to the new-born baby at the nearest health care facility. She was discharged after 6 h postnatally,*

*As she was working with the mother, she never transferred basic postnatal skills for the women and baby. Community-based postnatal care skill was never transferred either through training, health education, counselling, and referral system.*

The above-mentioned case reflects an absence of the attributes of community-based postnatal care.

- Identification of antecedents
  Antecedents are defined as events or incidents that must occur prior to the occurrence of the concept (Walker & Avant 2013). They are the determinants of the concept, things that cause a concept or risk factors of the concept ‘community-based postnatal care’, it emerged that the following are the necessary conditions that must be satisfied before the enhancement of community-based postnatal care. Antecedents assisted the researchers in identifying the underlying assumptions about the concept ‘community-based postnatal care’.
  The following are the antecedents as identified by the researchers:
  ■ A primary caregiver who had once had and successfully raise the baby
  ■ Attitudes of primary caregivers towards health care providers
  ■ Willingness of primary caregivers to change on harmful practices
  ■ Awareness of linkages of primary caregivers with health care facilities, especially community health care workers.
  ■ Knowledge and skills of basic/indigenous postnatal care and basic counselling.
  ■ Awareness on available referral resources.
- Identification of the consequences
  Consequences are the events or incidents that occur because of the occurrence of the concept and that often stimulate new ideas for research regarding new concepts (Walker & Avant 2013). In the case of concept ‘community-based postnatal care’, the identified processes are implemented to accomplish specific outcomes, which were to reduce or minimise maternal and neonatal deaths. The possible consequences of community-based postnatal care are:
  ■ Confident and competent postnatal woman who can do self-care to anticipate and identify potential complications
  ■ Improved quality of life for postnatal woman and the baby
  ■ No development of complications to mother and baby during the postnatal period
  ■ No maternal and neonatal morbidity and mortality within the community
  ■ Adherence to the attendance of postnatal care and antenatal care (PNC) visits by postnatal women
  ■ Definition of empirical referents
  Empirical referents are defined as classes or categories of actual phenomena that by existence or presence demonstrate the occurrence of the concept itself (Walker & Avant 2013). The empirical referents have been identified from the results on experiences of primary caregivers regarding the early discharge postnatal period. These empirical referents of the community-based postnatal care were measured through the following:
  ■ Self-care management of early postnatal care activities (for the mother and baby)
  ■ Early detection and early management of early postnatal complications
  ■ Early referral of postnatal complications

## Recommendations

In terms of midwifery practice, it is recommended that the empirical referents of community-based care be integrated within the midwifery guidelines to measure the concept. When the empirical referents of the concept are understood, self-care (early detection, early management and referral) during early postnatal care will be enhanced. Midwives are recommended to engage and involve primary caregivers for the early detection of postnatal complications. It is also recommended that the results of concept analysis for community based be included in postnatal care guidelines for the provision of quality postnatal care. Community participation in postnatal health education and counselling session as per community-based postnatal care is highly recommended. Postnatal care information should be integrated during the first antenatal care visits up until the 4th antenatal care (ANC) visits with primary caregivers’ engagement through health education and counselling antenatal.

## Conclusion

Challenges in the postnatal care units demanded the use of community-based care to facilitate self-care and active participation of primary caregivers during the postnatal period. The study elucidates the meaning of the concept community-based postnatal care. The analysis also led to the development of antecedents, attributes, model cases and consequences of the concept. Lastly, the empirical referents were also highlighted and discussed. Concept analysis is a part of knowledge development. All the clarified concepts can be used in midwifery practice, more especially the postnatal care period. The concept community-based care would foster independence, confidence and respectful relationship between the primary caregivers and the health care facilities staff. There would also be a partnership and collaboration in decision making between the midwives (community health care workers) and primary caregivers during the community-based care.
